# Rostafuroxin, the inhibitor of endogenous ouabain, ameliorates chronic undernutrition-induced hypertension, stroke volume, cardiac output, left-ventricular fibrosis and alterations in Na^+^-transporting ATPases in rats

**DOI:** 10.1016/j.jmccpl.2024.100281

**Published:** 2024-12-28

**Authors:** Amaury Pereira-Acácio, João P.M. Veloso-Santos, Camile O. Silva-Rodrigues, Debora Mello, Danilo S. Alves-Bezerra, Glória Costa-Sarmento, Humberto Muzi-Filho, Carlla A. Araújo-Silva, Jarlene A. Lopes, Christina M. Takiya, Sergian V. Cardozo, Adalberto Vieyra

**Affiliations:** a Graduate Program of Translational Biomedicine, Grande Rio University/UNIGRANRIO, Duque de Caxias, Brazil; b Leopoldo de Meis Institute of Medical Biochemistry, Federal University of Rio de Janeiro, Rio de Janeiro, Brazil; c Grande Rio University/UNIGRANRIO, Duque de Caxias, Brazil; d National Center for Structural Biology and Bioimaging/CENABIO, Federal University of Rio de Janeiro, Rio de Janeiro, Brazil; e Carlos Chagas Filho Institute of Biophysics, Federal University of Rio de Janeiro, Rio de Janeiro, Brazil

**Keywords:** Chronic undernutrition, Hypertension, Endogenous cardiotonic steroids, Rostafuroxin, Cardiac sodium ATPases

## Abstract

Our aim has been to investigate the effect of Rostafuroxin, an inhibitor of endogenous cardiotonic steroids (EO/CTS), on cardiac structure and function and cardiac Na^+^ transport in undernourished hypertensive Wistar rats, and to determine whether chronic undernutrition is a modifiable risk factor for cardiovascular-kidney-metabolic (CKM) syndrome. Echocardiographic studies evaluated stroke volume cardiac output, ejection fraction, mitral valve early diastolic blood flow/late diastolic blood flow (E/A) ratio, and right renal resistive index. The cardiomyocyte area and collagen infiltration of cardiac tissue were investigated, as also the activities of the cardiac ouabain-sensitive (Na^+^+K^+^)ATPase ((Na^+^+K^+^)ATPase Sens) and ouabain-resistant Na^+^-ATPase (Na^+^-ATPase Res). Undernourished hypertensive rats presented tachycardia, reduced stroke volume, decreased cardiac output, preserved fractional shortening and ejection fraction, unmodified mitral valve E/A ratio, and increased right renal resistive index. Cardiomyocyte size decreased and intense collagen infiltration had occurred. The (Na^+^+K^+^)ATPase Sens activity decreased, whereas that of Na^+^-ATPase Res increased. Rostafuroxin selectively modified some of these echocardiographic and molecular parameters: it increased stroke volume and cardiac output and prevented histopathological alterations. The drug decreased and increased the activities of (Na^+^+K^+^)ATPase Sens and Na^+^-ATPase Res, respectively, in normonourished rats, and the opposite trend was found in the undernourished group. It is concluded that chronic undernutrition in rats can provoke structural, functional, histological, and molecular cardiovascular alterations that, with the simultaneous changes in renal parameters described in this and in previous studies, configure an undescribed type of CKM syndrome. The data also demonstrate that the blockade of EO/CTS ameliorates stroke volume and cardiac output, thus preventing or delaying the worsening of the syndrome.

## Highlights

• Chronically undernourished and hypertensive rats develop heart failure.
• Undernourished rats present decreased cardiac output and stroke volume.
• Undernourished rats have decreased cardiomyocyte size and collagen infiltration.
• Increased renal artery resistive index reveals compromised kidney function.
• Rostafuroxin-sensitive mechanisms participate in anomalous cardiac sodium transport.

## Graphical abstract

**Figure:**
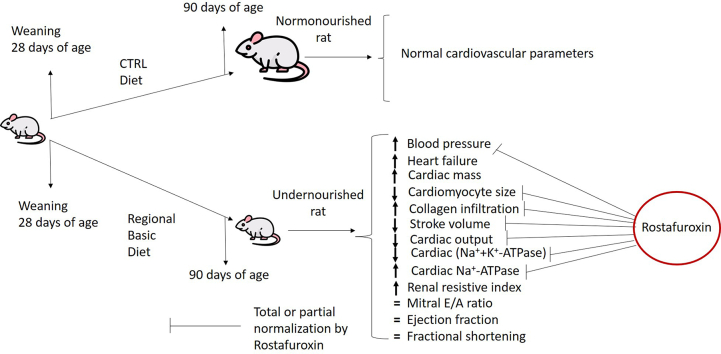

## 1 Introduction

The confluent association between metabolic risk factors, chronic kidney disease, and cardiovascular disease is conceptualized as a cardiovascular-kidney-metabolic (CKM) syndrome, which represents a growing and high-impact challenge for clinical and public health nowadays [1]. The systemic metabolic disturbances frequently associated with the CKM syndrome are diseases associated with an altered nutritional state, such as obesity, type 2 diabetes, and dyslipidemias [2], with dysfunctional visceral adipose tissue playing a central role in the ensemble of pathophysiological events [3].

While, until present, there has been no direct relationship between CKM syndrome and chronic undernutrition, it is crucial to understand that undernutrition can affect the kidney [4] and heart [5]. Chronic undernutrition induced by the Regional Basic Diet (RBD) mirrors those utilized in impoverished regions worldwide. This diet was formulated from the epidemiological observations of the alimentary habits of populations inhabiting rural areas in Northeast Brazil [6], which are similar to those widely encountered in different regions of the Middle East and America [[7], [8], [9]]. This research has global implications for understanding and addressing undernutrition-related health challenges.

In previous studies, we have demonstrated that the chronic ingestion of RBD provokes hyperactivity of the local renin-angiotensin-aldosterone system (RAAS) in the heart, loss of renal function with decreased plasma and urinary urea, and decreased of plasma albumin [10]. Undernutrition causes hypertension as a result of activation of the signaling coupled to type 1 Angiotensin II (Ang II) receptors in the kidney [11,12], inhibition of (Na^+^+K^+^)ATPase Sens in the left ventricle [11] and proximal tubules [12], with participation of endogenous cardiotonic steroids (CTS) [13].

CTS are molecules produced by both the hypothalamus and the adrenal gland [14,15]. They are released into the bloodstream and bind to (Na^+^+K^+^)ATPase Sens in different tissues and organs, inhibiting its activity and activating several signaling pathways. This promotes changes in renal Na^+^ handling and hypertension [16,17].

Our present work evaluates the potential of Rostafuroxin, an inhibitor of CTS [18], on cardiac structure and function, and on cardiac Na^+^ transport in hypertensive undernourished rats. This research also aims to determine whether chronic undernutrition is a modifiable risk factor for CKM, offering hope for potential interventions.

## 2 Materials and methods

### 2.1 Ethical considerations

The Committee for Ethics in Animal Experimentation of the Federal University of Rio de Janeiro approved the experimental procedures under protocol A03/23-066-21. The execution of these procedures adhered to the Committee's Guidelines, which align with the Uniform Requirements for Manuscripts Submitted to Biomedical Journals established by the International Committee of Medical Journal Editors. The animal study has been registered following the ARRIVE guidelines [19].

### 2.2 Diets

RBD is made up of the following ingredients (g/g%): beans, 18.3; manioc flour, 64.8; jerked meat, 3.7; and sweet potatoes, 12.8 [6]. These ingredients underwent cooking, dehydration at 60°C, and subsequent pulverization. The resulting diet has the centesimal composition (g/g%): protein, 8; carbohydrate, 69; lipid, 0.8; Na^+^, 0.19 ± 0.002; fiber, 8. Furthermore, the diet is deficient in protein quality, with most of the protein coming from beans. However, the energy supply adequacy is slightly higher (approximately 316 kcal/100 g dry weight) compared to the control diet (CTRL) (approximately 280 kcal/100 g). The CTRL diet was a regular chow from Neovia Nutrição e Saúde Animal (Contagem, Brazil) (g/g%: protein, 23; carbohydrate, 50; lipid, 9; Na^+^, 0.27 ± 0.005). The control diet was supplemented with vitamins to meet AIN-93G requirements [20], whereas the RBD lacked supplementation. The Na^+^ content of both diets was determined by flame photometry; this control being repeated each time a new RBD preparation or CTRL diet bag was used. The difference between the mean values mentioned above was assessed by unpaired Student's *t*-test (*t*=16.58; P<0.0001).

### 2.3 Experimental groups

Wistar male rats were weaned at 28 days of age and divided randomly into 2 cohorts until they reached 90: one group was provided with the RBD, while the other group received the CTRL diet. Drinking water was made available ad libitum to both groups. At 60 days, the 2 groups were further subdivided into 2 additional subgroups, one receiving Rostafuroxin (Rosta: 1 mg/kg body mass diluted in 99% ethanol; Aobious Inc., Gloucester, MA) and the other receiving an equivalent volume of water. As a result, the 4 experimental groups comprised CTRL, CTRL + Rosta, RBD, and RBD + Rosta. On day 90, the rats were weighed after the echocardiographic recordings and immediately before euthanasia by decapitation.

### 2.4 Measurements of systolic blood pressure and heart rate

Systolic blood pressure and heart rate were assessed at day 90 by the tail-cuff plethysmography method (model V2.01; Insight, Ribeirão Preto, Brazil) in conscious rats [21]. Despite its limitations, the method has advantages and allows comparison of values recorded at the same time [22,23]. The rats were trained and acclimated in a heated chamber in the preceding days and before the measurements [12]. The chamber's high temperature – higher than room temperature – under infrared light pointing towards the tail is required to ensure increased blood flow and the accuracy of the plethysmographic recording. The sphygmomanometer was automatically inflated and deflated, and the transducer signals were also automatically collected. The recorded values were the average of 5 determinations.

Heart rate was recorded in 2 experimental conditions: (a) with the rats awake during plethysmographic blood pressure measurements, and (b) with rats anesthetized with isoflurane during echocardiographic studies. This second recording indicated when the analysis began (350–450 bpm; see subsection 2.5).

### 2.5 Echocardiography

Echocardiography recordings were acquired at day 90 using Vevo 2100 ultrasound equipment (Fujifilm VisualSonics® Inc., Toronto, Canada) and an MS250 transducer (13–24 MHz). Under general anesthesia (2.5% isoflurane, 2 ml per litre O_2_), animals were submitted to thoracotomy and abdominal shaving with depilatory cream (Veet®, Reckitt Benckiser Health Comercial Ltda, São Paulo, Brazil) to access the heart and kidney. Animals were placed in supine positions for the imaging acquisition. A controlled heart rate between 350 and 450 bpm was used for cardiac evaluation. The parasternal long (PLAX), short (SAX) axis, and apical 2-chamber views were used to access heart geometry and function.

Analyses were performed offline using Vevo LAB (Fujifilm VisualSonics®, Inc.). Left ventricular end-diastolic and end-systolic volumes (LVDV, LVSV), ejection fraction (EF), stroke volume (SV), and cardiac output (CO) were calculated using Simpson's rule. Left ventricular end-diastolic, end-systolic diameters (LVDD, LVSD), and fractional shortening (FS) were calculated using M-Mode. Also, using the apical 2 chamber view, the mitral valve early diastolic blood flow/late diastolic blood flow (E/A) ratio was obtained. American Society of Echocardiography recommendations [24] were followed for the data analysis and interpretation. Immediately after the echocardiographic acquisition, renal B-mode cine loops of a transversal section of the right kidney were recorded. Color Doppler was used to identify the right renal artery; then, Doppler analysis was performed, placing the sample volume in the right renal artery. Peak systolic velocity (RRA PSV) and end-diastolic velocity (RRA EDV) were measured, and the renal resistive index (RRI) was automatically calculated.

### 2.6 Measurement of cardiomyocyte size (area) and cardiac collagen content

Cardiomyocyte size was quantified by immunofluorescence using pan-cadherin (intercalated discs staining) and wheat germ agglutinin lectin (cardiomyocyte membranes labeling) in paraffin sections (6 μm thick), according to [25]. After dewaxing sections in xylene, they were rehydrated through a decreasing ethanol series and were submitted to a protein bath constituted by 5% bovine serum albumin, fraction V (Sigma-Aldrich, Saint Louis, MO) in phosphate saline buffer (PBS at pH 7.4) for 18 h (4°C), to block autofluorescence-induced by histological processing. Sections were then permeabilized with 0.5% Triton X-100 in PBS for 15 min and submitted to heat-mediated antigen retrieval in a steam-cooker (96°C), in TRIS-EDTA buffer (10 mM TRIS base, 1 mM EDTA; pH 9.0) for 20 min. After cooling, histological sections were incubated with a blocking solution containing 5% normal sheep serum (Sigma-Aldrich), 5% bovine serum albumin, fraction V, 0.005% bovine skin gelatin (Sigma-Aldrich), 0.005% Triton X-100 (Sigma-Aldrich), 0.025% Tween 20 (Sigma-Aldrich) in PBS, for 30 min (at room temperature), to block nonspecific binding of immunoglobulins to tissue proteins. The sections were then submitted to another blocking step using a solution containing 0.2 M glycine solution in PBS for another 30 min. To block endogenous biotin, sections were rapidly immersed in PBS and were incubated with streptavidin and biotin solutions from the streptavidin/biotin blocking kit (Vector Laboratories Inc., Newark, CA) following the manufacturer's instructions. After washing with PBS (pH 7.4), sections were incubated with pan-cadherin antibody (EPR1792Y rabbit monoclonal, Abcam, Cambridge, UK; dilution 1 μg/ml) overnight at 4°C. After washing in a 0.25% Triton X-100 in PBS solution (PBS-T), sections were incubated with anti-rabbit IgG (whole molecule) F(ab')_2_ fragment–Cy3 conjugated antibody (Sigma-Aldrich; dilution 1:400) for 1 h, followed by PBS-T baths. Another blocking step was performed using 0.2 M glycine solution in PBS for 30 min and then incubated with biotinylated wheat germ agglutinin (WGA) from Vector Laboratories (dilution 1:100) for 1 h at room temperature. Following this, streptavidin conjugated with fluorescein (SA-5001-1, Vector Laboratories; dilution 1:100) was incubated for 30 min, followed by nuclei staining using Hoechst 33342 (Thermo-Fisher, Waltham, MA; dilution 1 μg/ml) for 10 min. Both pan-cadherin and biotinylated WGA were diluted in 10 mM HEPES (pH 7.5), 0.15 M NaCl, 0.08% NaN_3_, and 0.1 mM CaCl_2_. After baths with PBS, nuclei were stained with Hoescht 33,342 (Thermo-Fisher Scientific, Waltham, MA; dilution 2 μg/ml in distilled water); sections were mounted with Vectashield mounting medium (Vector Laboratories). Imaging was performed using a Zeiss confocal laser scanning microscope LSM900 (Carl Zeiss AG, Oberkochen, Germany) using a 63× objective in a *Z*-stack, with maximum intensity projection for measurement of cardiomyocytes. Ten fields per condition were randomly obtained. The software Zen blue 3.9 (Zeiss) was used to measure cardiomyocyte size.

Collagen deposition was visualized in paraffin sections using a modified Picrosirius red (PSR) staining procedure [26]. In brief, paraffin sections (8 μm thick) were dewaxed and hydrated, washed with an aqueous 0.2% phosphomolybdic acid solution for preventing nuclear staining and then stained with a 0.1% Sirius red (Sigma-Aldrich) in saturated picric acid solution for 90 min, washed in 70% ethanol, clarified in xylene and mounted with Entellan™ (Sigma-Aldrich). For collagen deposition quantification, images were taken using an image capture and analysis system consisting of a light microscope coupled to a digital camera (Evolution VR Cooled Color 13 bits, Media Cybernetics, Rockville, MD). The interface capture software used was Q-Capture 2.95.0, version 2.0.5 (Silicon Graphics Inc., Sunnyvale, CA). High-resolution images (2048 × 1536 pixel buffer) were transmitted to a color LCD monitor in TIFF format and digitized. The images were captured after calibration of the appropriate color and contrast parameters and remained constant for each type of 10 random images of per tissue section. A 40× objective was used to get images avoiding areas containing vessels or endocardia/pericardia. Collagen fibers/fibrils present in the heart area appeared red. Only the interstitial collagen fibers were considered and quantified (collagen surface density or % collagen) [27] using the ImageJ software [http://imagej.nih.gov/ij].

### 2.7 Isolation of cardiomyocyte plasma membranes

Cardiomyocyte plasma membranes were isolated through sequential centrifugation, following the method outlined in Dostanic et al. [28]. Hearts were removed following euthanasia, carefully dried with filter paper, weighed, kept on ice, and dissected to isolate the left ventricle, which was then minced into small fragments. These fragments were suspended in an isotonic solution containing 250 mM sucrose, 1 mM imidazole (pH adjusted to 7.6 using Tris), and 1 mM EDTA. Mechanical homogenization of the preparations was carried out at 4°C using a Potter Elvejhem homogenizer with a Teflon pestle (with 5 periods of 1 min at 1,700 rpm). The resulting suspension underwent centrifugation at 1,669 × *g* for 15 min at 4°C, and the resulting supernatant was centrifuged at 115,000 × *g* for 60 min at 4°C. The final sediment was suspended in 250 mM sucrose and stored in a −80°C freezer. Protein concentration was determined using the Folin method [29].

### 2.8 Measurement of ouabain-sensitive (Na^+^+K^+^)ATPase Sens and ouabain-resistant Na^+^-ATPase Res activities

The ouabain-sensitive (Na^+^+K^+^)ATPase Sens and the ouabain-resistant, furosemide-sensitive Na^+^-ATPase Res activities were assessed by measuring the inorganic phosphate (P_i_) release during ATP hydrolysis. These activities were measured in triplicate following the protocol outlined by Pereira-Acácio et al. [13]. The activities were determined by the difference between the measurements obtained in the absence and presence of 2 mM ouabain for (Na^+^+K^+^)ATPase Sens and in the absence and presence of 2 mM furosemide for Na^+^-ATPase Res.

### 2.9 Statistical analysis

The results are presented as mean ± standard error of the mean (SEM). The group differences were assessed through 2-way ANOVA followed by Bonferroni's test. Statistical analyses were performed using GraphPad Prism 8 software (version 8.02, GraphPad Software, Inc., Boston, MA). Specific comparisons between groups not covered by 2-way ANOVA were carried out using unpaired *t*-test and described in the text. In the case of histomorphometric studies (semiquantitative data) the group differences were assessed using two-way ANOVA mixed effect. Statistical significance was defined at P<0.05.

## 3 Results

### 3.1 Chronically undernourished rats develop hypertension and present with increased heart rate

**Fig. 1:**
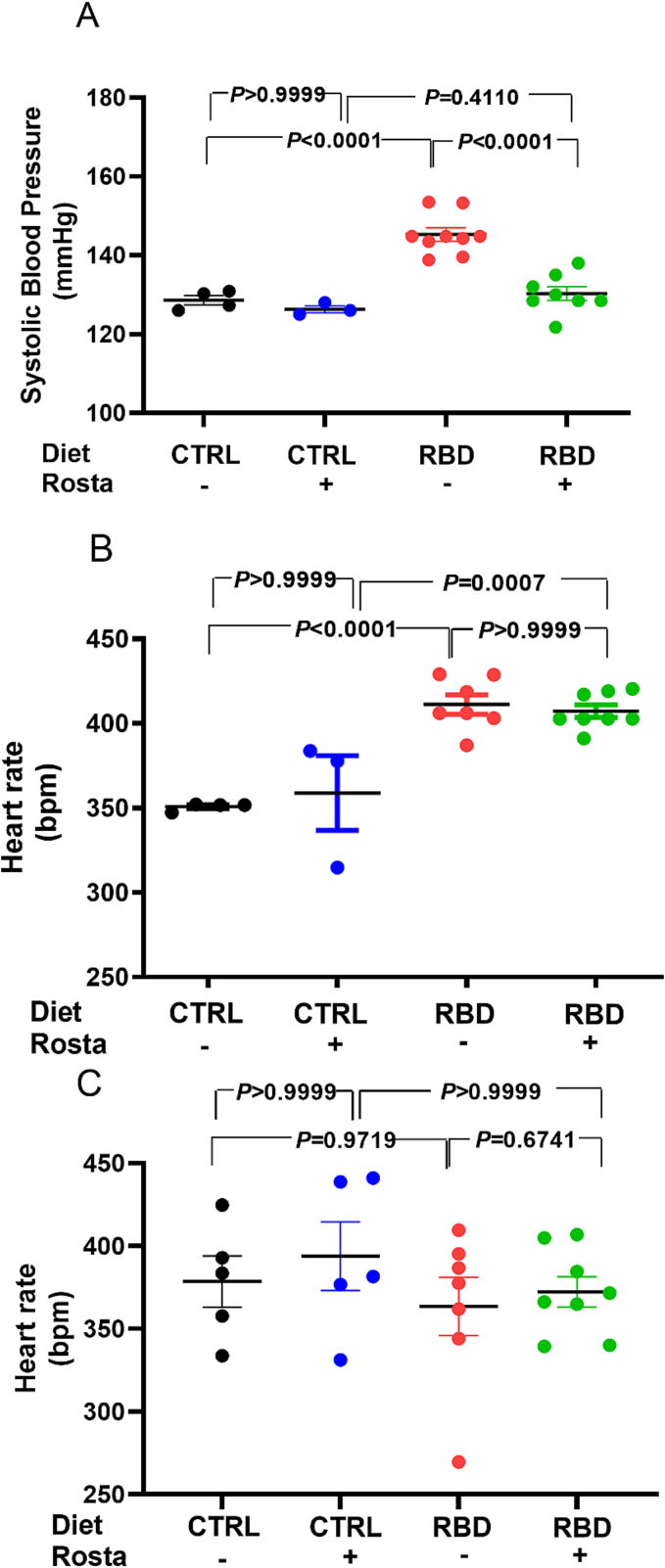
Assessment of systolic blood pressure and heart rate at 90 days of age in undernourished and normonourished rats: effect of Rostafuroxin. (A) Systolic blood pressure. (B) Heart rate in conscious rats. (C) Heart rate in anesthetized rats. Diets and treatment or not with Rostafuroxin (Rosta) are indicated on the abscissae. Scatter plots show mean ± SEM. Differences were assayed using two-way ANOVA followed by Bonferroni's test; P values are indicated within the panels. In (A), n = 4, 3, 9, and 8 for CTRL, CTRL + Rosta, RBD, and RBD + Rosta, respectively. In (B), n = 4, 3, 7, and 8 for CTRL, CTRL + Rosta, RBD, and RBD + Rosta, respectively. In (C), n = 5, 5, 8, and 8 for CTRL, CTRL + Rosta, RBD, and RBD + Rosta, respectively. In all cases, n corresponds to one rat. The data of panel A are reproduced from Pereira-Acácio et al. [13] under the terms of Creative Common License CC BY 4.0.

Fig. 1A demonstrates that chronically undernourished rats became hypertensive at the emerging adulthood [30] after 62 days of exposure to the deficient RBD compared to the normonourished animals. The RBD group progressively developed hypertension (145 versus 129 mmHg at day 90) [13], which was prevented by the administration of Rostafuroxin.

Fig. 1B, C shows the different heart rate profiles depending on whether the rats were awake or anesthetized. While chronic malnutrition leads to tachycardia in the former (Fig. 1B), no differences were observed between the 4 groups under anesthesia (Fig. 1C). Rostafuroxin had no effect on heart rate, regardless of the rats' state of consciousness.

### 3.2 Increased cardiac mass/body mass ratio in undernourished rats

**Fig. 2:**
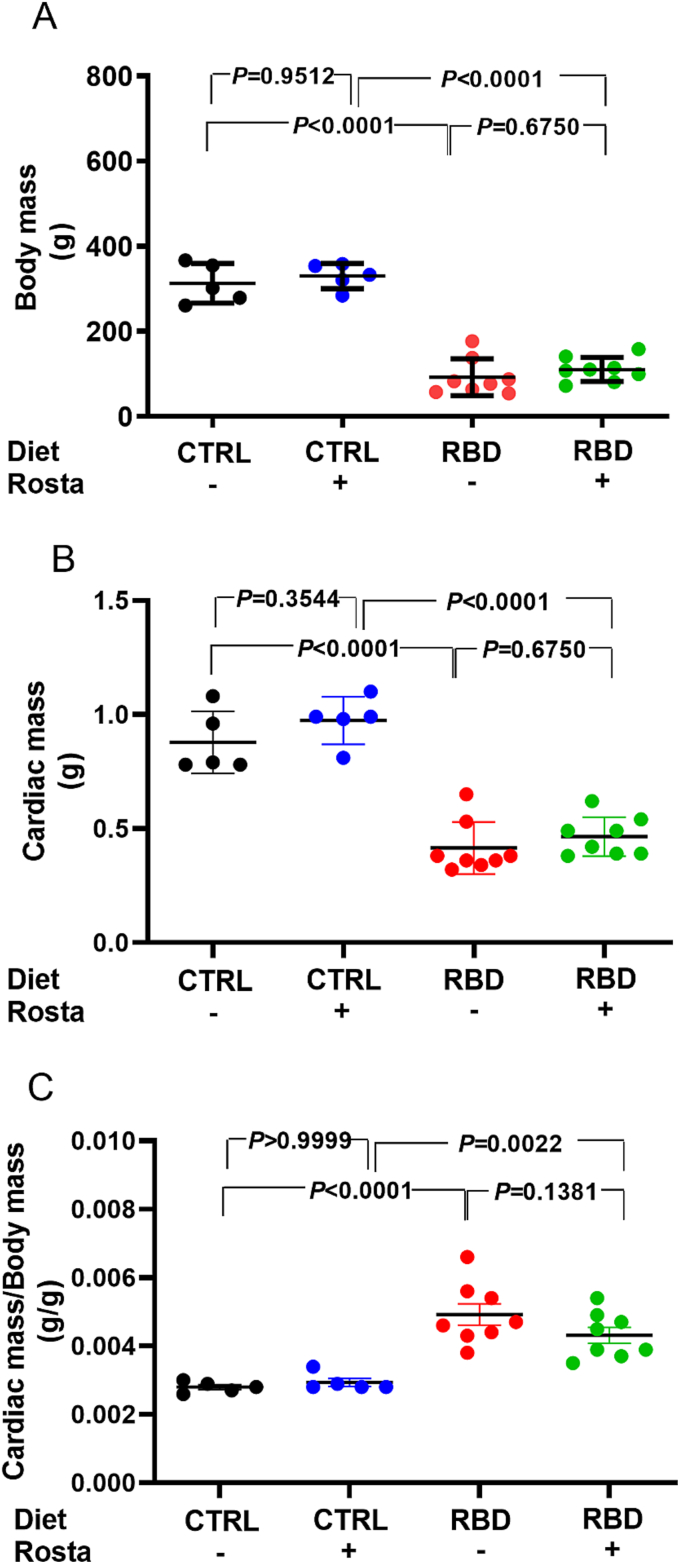
Assessment of body mass, cardiac mass, and cardiac mass/body mass in undernourished and Rostafuroxin-sensitive hypertensive rats: comparison with normonourished animals. (A) Body mass. (B) Cardiac mass. (C) Cardiac mass/Body mass. Diets and treatment or not with Rosta are indicated on the abscissae. Scatter plots show mean ± SEM. Differences were assayed using two-way ANOVA followed by Bonferroni's test; P values are indicated within the panels. In (A), (B), and (C), n = 5, 5, 8, and 8 for CTRL, CTRL + Rosta, RBD, and RBD + Rosta, respectively. In all cases, each n corresponds to one rat.

The 90 days old-undernourished (RBD) rats presented an average body mass of ∼70% lower than that of the CTRL group (Fig. 2A). Despite its antihypertensive action (Fig. 1A), Rostafuroxin did not modify body mass evolution in either of the groups. The cardiac mass (Fig. 2B) followed a similar trend to the body mass, with a ∼50% reduction in the RBD group compared to CTRL and, again, with no effect of Rostafuroxin. When the heart mass/body mass ratio was calculated, the results revealed that the undernourished rats presented a value that was approximately twice that encountered in the normonourished animals (Fig. 2C) and, as expected, without the effect of Rostafuroxin.

**Fig. 3:**
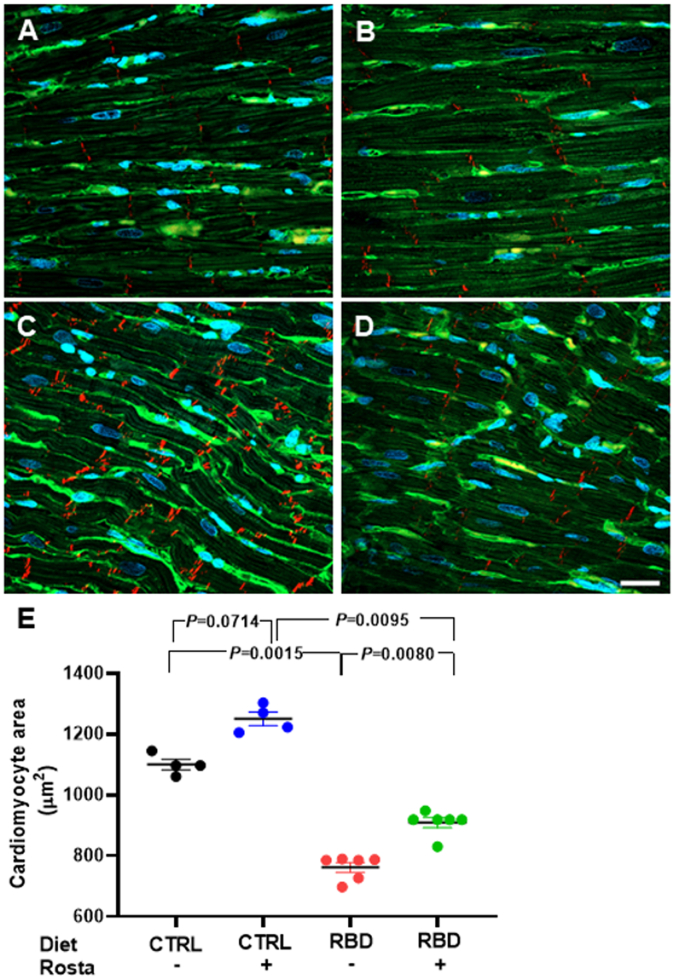
Measurement of cardiomyocyte area in normonourished and undernourished rats: effect of Rostafuroxin. (A, B, C, and D) Representative immunofluorescence images of pan-cadherin and wheat germ agglutinin lectin of CTRL, CTRL + Rosta, RBD, and RBD + Rosta, respectively. (E) Quantification of cardiomyocyte area. Diets and treatment or not with Rosta are indicated on the abscissa. Scatter plots show mean ± SEM; n = 4, 4, 6, and 6 for CTRL, CTRL + Rosta, RBD, and RBD + Rosta. In all cases, n corresponds to slices from different rats. Differences were assayed using two-way ANOVA mixed effect followed by Bonferroni's test; P values are indicated within the panels.

**Fig. 4:**
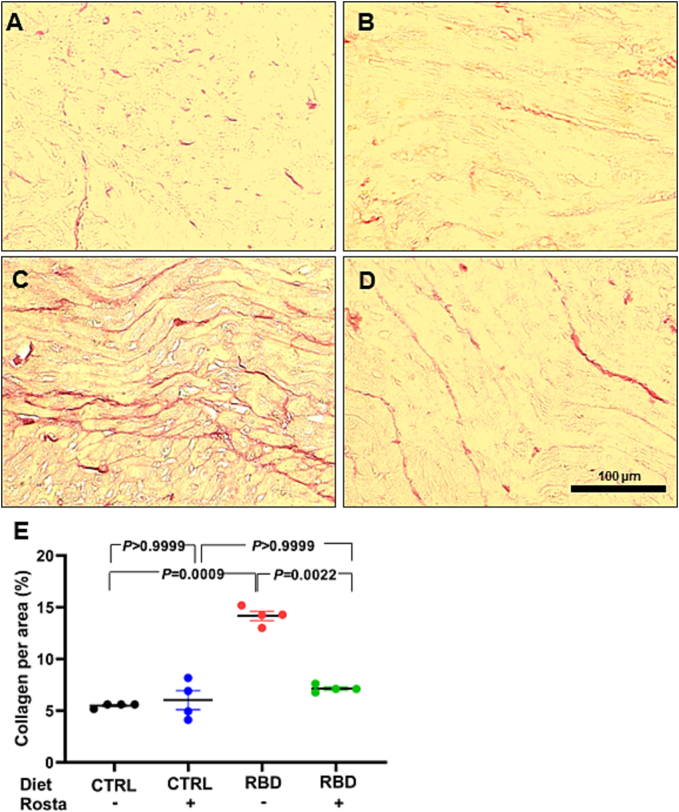
Measurement of collagen content in normonourished and undernourished rats: effect of Rostafuroxin. (A, B, C, and D) Representative Picrosirius red-stained images of CTRL, CTRL + Rosta, RBD, and RBD + Rosta, respectively. (E) Quantification of collagen content. Diets and treatment or not with Rosta are indicated on the abscissa. Scatter plots show mean ± SEM; n = 4, 4, 4, and 4 for CTRL, CTRL + Rosta, RBD, and RBD + Rosta. In all cases, n corresponds to slices from different rats. Differences were assayed using two-way ANOVA mixed effect followed by Bonferroni's test; P values are indicated within the panels. (For interpretation of the references to color in this figure legend, the reader is referred to the web version of this article.)

Fig. 3, Fig. 4 present histological results intended to compare cardiomyocytes' size and collagen amount in CTRL and RBD rats treated or not with Rostafuroxin. The greater number of intercalated discs detected in immunofluorescence (pan-cadherin) in the DBR group indicates a reduction in the area of cardiomyocytes. Increased WGA labeling highlights microcirculation due to decreased cardiomyocyte size (Fig. 3). Thus, immunofluorescence images of double-stained techniques, using pan-cadherin antibody to highlight intercalated discs and WGA lectin to show cardiomyocyte membranes, demonstrated that RBD rats had a decreased size (762 ± 16 μm^2^) compared to CTRL, CTRL + Rosta and RBD + Rosta animals (1100 ± 17 μm^2^, 1250 ± 22 μm^2^, and 909 ± 17 μm^2^, respectively). Associated with cardiomyocytes decreased size in RBD rats, the quantification of PSR stained sections (Fig. 4) shows a noticeable increment in the amount of collagen in the heart of RBD rats (14.2 ± 0.4%) compared to CTRL, CTRL + Rosta and RBD + Rosta animals (5.5 ± 0.1%, 6.0 ± 0.9%, and 7.1 ± 0.2%, respectively), likely responsible for the augmented cardiac mass/body mass ratio (Fig. 2C).

### 3.3 Cardiac function assessment in undernourished rats

**Fig. 5:**
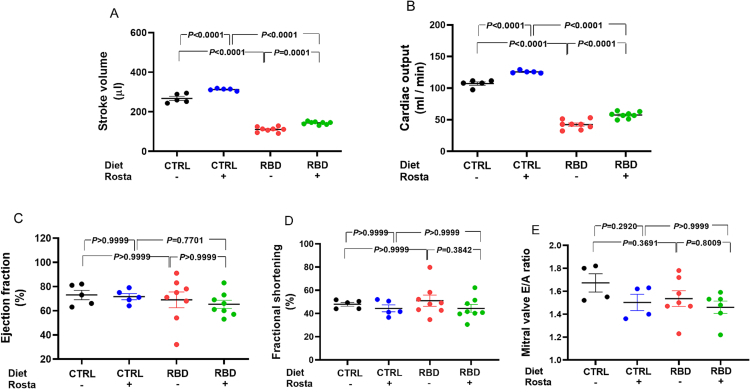
Comparison of stroke volume (SV), cardiac output (CO), ejection fraction (EF), fractional shortening (FS), and mitral valve E/A ratio from normonourished and undernourished rats: effect of Rostafuroxin. Diets and treatment or not with Rostafuroxin are indicated on the abscissae. Scatter plots show mean ± SEM. Differences were assayed using two-way ANOVA followed by Bonferroni's test; P values are indicated within the panels. The number of rats (n) was 5, 5, 8, and 8 for CTRL, CTRL + Rosta, RBD, and RBD + Rosta, respectively (in the study of all echocardiographic parameters). In all cases, each n corresponds to a different rat.

Fig. 5 compares the stroke volume (SV), cardiac output (CO), ejection fraction (EF), fractional shortening (FS), and mitral valve E/A ratio of CTRL and RBD rats. It also shows the effects of Rostafuroxin administration. The SV (A) was ∼60% lower in RBD rats and CO (B) decreased by >50% in RBD rats compared to CTRL, and this parameter was increased in both groups with the Rostafuroxin treatment. Notably, the response to the drug was more intense in RBD rats (25% versus 15%, and 36% versus 17% in the case of SV and CO, respectively) when average values were compared.

The EF is preserved in the undernourished rats (∼65%) and is also insensitive to Rostafuroxin (C), as well as the FS (∼50%) (Fig. 5C, D). Finally, the left ventricular diastolic function was evaluated as the ratio between the early diastolic blood flow (wave E) and the late diastolic blood flow (wave A) through the mitral valve [31,32]. Panel E shows that the mitral valve E/A ratio is similar in the 4 groups ranging from 1.5 to 1.7.

### 3.4 Increased renal resistive index in undernourished rats

**Fig. 6:**
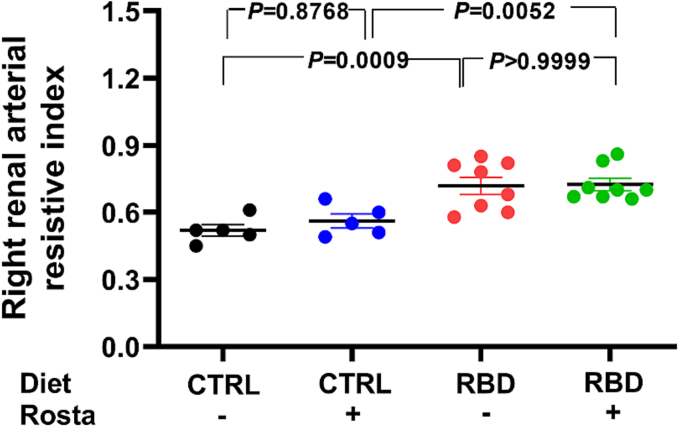
Right renal arterial resistive index (RRI). RRI was calculated as the difference between the peak systolic blood velocity (PSV) and the end diastolic blood velocity (EDV) divided by the peak systolic blood velocity ([PSV - EDV] / PSV). Diets and treatment or not with Rostafuroxin are indicated on the abscissa. Scatter plots show mean ± SEM. Differences were assayed using two-way ANOVA followed by Bonferroni's test; n = 5, 5, 8, and 8 for CTRL, CTRL + Rosta, RBD, and RBD + Rosta, respectively. In all cases, each n corresponds to a different rat. P values are indicated within the panels.

The renal resistive index (RRI) allows evaluating the complex relations between systemic and renal circulation in physiological and pathological conditions [33]. Since RRI is an important marker in predicting heart failure progression [34], we investigated whether RRI is modified in RBD rats (Fig. 6). The mean RRI was ∼40% higher in the undernourished RBD group than the normonourished CTRL rats, without any effect of Rostafuroxin in both groups.

### 3.5 Modifications in cardiac left ventricle Na^+^-transporting ATPases

**Fig. 7:**
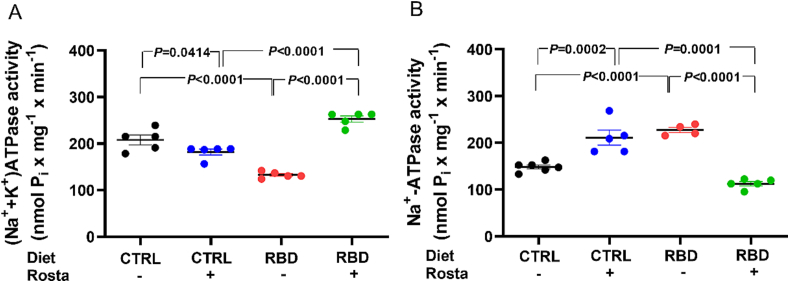
Different profiles of chronic undernutrition-induced alterations in left ventricle Na^+^-transporting ATPases: Opposite effects of Rostafuroxin. Diets and treatment or not with Rostafuroxin are indicated on the abscissae. (A) Ouabain-sensitive (Na^+^+K^+^)ATPase Sens. (B) Ouabain-resistant Na^+^-ATPase Res. Scatter plots show mean ± SEM. Differences were assayed using two-way ANOVA followed by Bonferroni's test. (A) n = 5 (all groups). (B) n = 6, 5, 4, and 5 for CTRL, CTRL + Rosta, RBD, and RBD + Rosta, respectively. Each n corresponds to a microsomal preparation from 3 (CTRL) and 6 (RBD) hearts of different rats. P values are indicated within the panels. Specific comparisons between groups not covered by two-way ANOVA are indicated in the text.

Fig. 7 depicts the results obtained with the 2 Na^+^-transporting ATPases. In the case of the ouabain-sensitive (Na^+^+K^+^)ATPase Sens, there was a decrease of ∼40% in its activity compared to CTRL (Fig. 7A). Rostafuroxin had a significant – though small – effect in the normonourished CTRL rats, but doubled the activity of the enzyme in the RBD. An additional statistical comparison revealed that the activity of the RBD + Rosta group was significantly upregulated above the levels encountered in untreated CTRL rats (*t*=3.609, P=0.0069).

In the case of the ouabain-resistant Na^+^-ATPase Res, its activity in the undernourished group was the mirror image of that encountered with the (Na^+^+K^+^)ATPase Sens: it increased ∼50% in the RBD group compared to CTRL (Fig. 7B). In addition, the response to Rostafuroxin was completely different in both ATPases because, in this case (i) significant upregulation was seen in the CTRL Rosta group (∼40%) in relation to untreated CTRL (*t*=4.146; P=0.0025), and (ii) a significant decrease of ∼50% was encountered when the RBD + Rosta rats were compared against the RBD rats. This reduction led the Na^+^-ATPase Res activity of the RBD + Rosta rats significantly below that of the CTRL (*t*=5.761; P=0.0003).

## 4 Discussion

One of the main findings of the study is the demonstration that hypertension, tachycardia, structural alterations of cardiomyocytes, accentuated decrease of SV (and CO), elevated RRI, and alterations in left-ventricular Na^+^-transporting ATPases, complete the cardiovascular component of the triad cardiovascular-kidney-metabolic syndrome (CKM) in chronically undernourished rats [[1], [2], [3], [4]]. This syndrome has been described in overweight/obese [2] but not in undernourished mammals until now. In addition, the simultaneous molecular alterations at the level of cardiac (this study) and renal [11,35] Na^+^-transporting ATPases leads us to propose that they are also part of this syndrome.

Myocardial infarction in RBD rats with chronic undernutrition is associated with upregulation of the propranolol-sensitive β-adrenergic receptors [10], suggesting that sympathetic activation could be a central molecular mechanism of the pathogenesis of the cardiac alterations here described [36], and that repeated non-lethal infarction episodes are behind the echocardiographic alterations in SV and CO observed in RBD rats in the present study (Fig. 5A, B). Even though the β-adrenergic activation in cardiac tissue could be considered a central adaptive mechanism tempting to compensate – via increased HR – (Fig. 1B) the undernutrition-induced lower pumping capacity of the organ (decreased SV and CO), other molecular mechanisms probably participate in the pathogenesis of CKM in RBD rats (for a review see [37]). The combination of these mechanisms and the β-adrenergic upregulation is probably crucial in the functional remodeling that culminates with decreased pumping capacity in undernourished rats.

Furthermore, the decreased size of cardiomyocytes and fibrosis in the RBD group also contributes to the functional remodeling of the contractile fibrillar machinery. Decreased collagen turnover and increased turnover of contractile proteins (revealed by decreased cardiomyocyte size) leads to interstitial fibrosis [38]. The same processes have been described in the thymus, in human undernutrition [39].

Even though the pumps are different, upregulation of Na^+^-ATPase Res found in our study may also contribute to the increase in fibrosis. In a model of chronic kidney disease (5/6th partial nephrectomy), an increase in cardiac (Na^+^+K^+^)ATPase Sens activity associated with tissue fibrosis was described, which decreases when the pump is inhibited [40,41]. In other studies, these observations add a new element to the kidney/heart communication in the CKM syndrome described herein. Finally, it deserves special mention that increased circulating EO/CTS stimulate collagen production in uremic cardiomyopathy [42] and upregulates myofibroblast differentiation in lung fibrosis [43] and in other pathological processes [44].

The partial reversal by Rostafuroxin of the impacts of undernutrition on the SV and CO, probably mediated by the molecular mechanisms discussed above, [43,44] suggest that the increasing stretch of the ventricle fibers before contraction is modulated by EO/CTS, i.e. by modulation of the signaling pathways linked to (Na^+^+K^+^)ATPase Sens [17,45]. Also at a molecular level, this means an influence on actomyosin ATPase kinetics, Ca^2+^ movements, and increasing fibril cross-bridges in the pre-power stroke [46], which is so far a novel observation regarding the physiological actions of endogenous digitalis.

The augmented RRI (∼30%) in RBD rats in comparison with the CTRL group indicates that vascular and parenchymal renal dysfunctions constitute key associated abnormalities in CKM [47], which are encountered here in a nutritional status characterized by chronic undernutrition. As demonstrated in previous studies, juvenile RBD rats develop several functional, structural, and molecular/signaling alterations (such as in the PKC and PKA activities ratio) that reveal a complex status of chronic kidney disease [11] with repercussion on the Rostafuroxin-sensitive bodily Na^+^ homeostasis [13].

In terms of the CKM syndrome, some of the studies above [11,13] revealed a central, similar molecular altered mechanism in the kidney: the downregulation of the ouabain-sensitive (Na^+^+K^+^)ATPase Sens, the ionic pump responsible for most of the cell's energy consumption, including the heart cells [48]. In the renal proximal tubule cells, RBD rats present an accentuated reduction (∼40%) of (Na^+^+K^+^)ATPase Sens activity [13,35] and the similar trend was found in the left ventricle (this study). Isoform-specific regulation of myocardial (Na^+^+K^+^)ATPase Sens participates in cardiac remodeling [49] in a complex process in which propranolol-sensitive β-adrenergic receptors as proposed 3 decades ago [50], and already discussed above [10]. These observations, taken as a whole, are indicative that the same pathological process involving (Na^+^+K^+^)ATPase Sens affects, at the same time, the heart and the kidney, as another example of synchronous organ dysfunction in the cardiorenal syndrome provoked by chronic undernutrition. The upregulation of the cardiac (Na^+^+K^+^)ATPase Sens above the CTRL levels by Rostafuroxin only in the RBD + Rosta group – not in the kidney [13] – is indicative that EO/CTS compounds are elevated in the plasma of RBD rats, have a selective target in the heart, and participate in the downregulation of the (Na^+^+K^+^)ATPase Sens-mediated cardiac Na^+^-pumping activity. The opposite response of the ouabain-resistant Na^+^-ATPase Res to Rostafuroxin in CTRL and RBD animals suggests that different EO/CTS-stimulated phosphorylation sites are involved. Finally, variations in the local RAAS would explain the opposite responses of Na^+^-ATPase Res to Rostafuroxin because in kidney (and possibly in heart): (i) Ang II-positive cells are higher in RBD; (ii) EO/CTS are regulated by the signaling pathways linked to type 2 Ang II receptors [35], which antagonizes the signaling events associated to type 1 Ang II receptors [51].

## 5 Conclusion

We have demonstrated the relationship between undernutrition and CKM and how it can be a cardiac risk factor for this syndrome. The results also point to selective actions of EO/CTS in several parameters of cardiac structure and functioning. In addition, Rostafuroxin improved cardiac function at least in part due to restoration of (Na^+^+K^+^)ATPase Sens activity.

## CRediT authorship contribution statement

**Amaury Pereira-Acácio:** Writing – review & editing, Writing – original draft, Validation, Methodology, Investigation, Formal analysis, Conceptualization. **João P.M. Veloso-Santos:** Writing – review & editing, Writing – original draft, Validation, Resources, Methodology, Formal analysis, Conceptualization. **Camile O. Silva-Rodrigues:** Writing – review & editing, Writing – original draft, Validation, Resources, Methodology, Formal analysis. **Debora Mello:** Writing – review & editing, Writing – original draft, Validation, Resources, Methodology, Investigation, Formal analysis, Conceptualization. **Danilo S. Alves-Bezerra:** Writing – review & editing, Writing – original draft, Visualization, Validation, Resources, Methodology. **Glória Costa-Sarmento:** Writing – review & editing, Writing – original draft, Validation, Resources, Methodology, Investigation. **Humberto Muzi-Filho:** Writing – review & editing, Writing – original draft, Validation, Resources, Methodology, Investigation, Formal analysis, Conceptualization. **Carlla A. Araújo-Silva:** Writing – review & editing, Visualization, Methodology, Investigation. **Jarlene A. Lopes:** Writing – review & editing, Visualization, Methodology, Investigation. **Christina M. Takiya:** Writing – review & editing, Visualization, Methodology, Investigation, Conceptualization. **Sergian V. Cardozo:** Writing – review & editing, Writing – original draft, Visualization, Supervision, Funding acquisition, Formal analysis, Conceptualization. **Adalberto Vieyra:** Writing – review & editing, Writing – original draft, Visualization, Validation, Supervision, Resources, Project administration, Investigation, Funding acquisition, Formal analysis, Conceptualization.

## Funding sources

This study was supported by the Brazilian National Research Council/10.13039/501100003593CNPq [grants 154249/2023-8, 123741/2023-8, 370753/2024-0, 311578/2019-5 and 409690/2023-6]; the Carlos Chagas Rio de Janeiro State Foundation/10.13039/501100004586FAPERJ [grants E-26/203.405/2023, E-26/201.434/2021, E-26/200.866/2021 and E-26/211.276/2021]; and the Brazilian Federal Agency for Support and Evaluation of Graduate Education/10.13039/501100002322CAPES [grants 88887.374390/2019-00 and 88887.634142/2021-00, and 88887.835146/2023-00]. The funding agencies had no involvement in study design; in the collection, analysis and interpretation of the data; in writing of the report; and in the decision to submit the article for publication.

## Declaration of competing interest

The authors declare no potential conflict of interest.

## Data Availability

The datasets generated and/or analyzed during the current study are available from the corresponding author on reasonable request.
